# Annotations

**Published:** 1902-05-03

**Authors:** 


### ANNOTATIONS.

### The Notification of Phthisis.

While feeling every sympathy with all reasonable attempts to control the spread of tuberculosis it is, we think, a matter on which the country may be distinctly congratulated that when the Halifax Corporation Bill recently came before the Committee of the House of Commons a clause which had been inserted with the object of rendering the notification of phthisis compulsory was disallowed. The case for notification was ably put by Dr. Neech, the medical officer of health, who showed that if infected houses were thoroughly disinfected they would cease to be a danger to others dwelling in them or to new tenants entering them, and that this systematic disinfection could only be insured by aid of compulsory notification. When, however, he touched upon the difficulty connected with the almost inevitable interference with the wage-earning capacity of those who are only slightly affected with the disease, were their malady made the subject of notification, he could only quote certain statements from America to the effect that inconvenience had not been caused to patients in certain parts where notification of phthisis had been in force. Against this we may suppose that the Committee brought to bear their common sense and their knowledge of human nature, for they disallowed the clause. When notification is enforced, provision must be made for the patients who will certainly be thrown out of employment, and for that the State is not yet prepared. In any case, we agree with what was said by the chairman of the Committee to the effect that if notification of phthisis is to be made compulsory this should be done by general and not by local order.

### Native Labour in the Tropics.

Travellers in the tropical East, noting the swarming population, and bearing in mind the extraordinary cheapness of human labour as measured in coin, are apt to regard "native labour" as an inexhaustible reserve which can be drawn upon as occasion may demand for all the varied kinds of work required in the development of these countries. Recent experience, however, in some of our new possessions has shown that the supply of native labour is by no means so inexhaustible as had been imagined, and that any scheme of development which hinges upon it becomes a much more complicated affair than it had seemed in the estimation of the earlier pioneers. In a paper read before a recent meeting of the Epidemiological Society on the subject of Infantile Mortality in the Tropics, Dr. Daniels showed that high as is the birth-rate in many tropical countries it is only just sufficient to compensate, and in some countries does not fully compensate for the very high death-rate which prevails. Thus there is but little power in the native races, so long as the present ratio between births and deaths continues to meet any large demand upon them, and the only way in which a continuous and reliable supply of the native labour which is so necessary for the development of tropical countries can be secured is by lessening the infantile mortality which prevails in them; for it is by the deaths among the children that the high mortality is maintained. Dr. Daniels said that in India the deaths in the first year of life were in few districts under 200 per 1,000 births, and in the Central Provinces reached 440. In Lagos and Freetown, Sierra Leone, the deaths in the first year formed between 35 and 40 per cent. of total deaths, while those of children between one and five years old formed another 35 per cent. of all deaths. In Central Africa no doubt European administration had abolished the fearful sacrifice of life connected with the slave trade, and had reduced the deaths from violence, but the aggregation of the natives on engineering works, and the overcrowding consequent on the adoption of European methods of housing, had led to an increase of tuberculosis and other ailments, so that the population showed a tendency to decrease. It was evident then that to ensure a permanent supply of labour in the tropics the infant mortality must be reduced. Importation of labour was but a temporary substitute.

### A Medical Boycott.

Members of the British Medical Association, many of whom, no doubt, consider that they belong to a quiet, steady-going, and respectable institution to which anything in the shape of trades-unionism is entirely foreign, will probably be interested in hearing to what depths a scientific body, indeed a branch of their own Association, may be dragged when run on trades-union lines and worked "for the advancement of the profession." It appears that in Australia there is an organisation called the Australian Natives' Association which, among other things, professes to provide medical attendance on terms which, while acceptable, or at any rate accepted by some of the medical men there, are disapproved of by others. We need not say anything as to the actual amount of the payment offered, because the different value of money in that part of the world takes away much of its significance. The real fight is over exactly the same point as troubles us here in regard to the clubs—namely, the wage limit. But how differently the fight is managed there and here! At the last meeting of the New South Wales Branch of the British Medical Association, which we are told was "one of the largest and most enthusiastic" of its gatherings, a resolution was passed in accordance with which, in the words of the *Australasian Medical Gazette*, no member of the branch "can take the position of medical officer to any branch of the Australian Natives' Association, and any medical man who takes such a position will not be met in consultation by any member of the New South Wales Branch of the British Medical Association." At a meeting of the Queensland branch also, resolutions having the same effect were passed, a further one in favour of extending the boycott to all who refused to conform with this ruling of the branch being held over until some "blackleg" should be reported. Now this is trades-unionism pure and simple, and this is the sort of use to which we may expect the Association in England to be turned if some of its members have their way.

